# METHODOLOGICAL CHALLENGES WITH VOLATILE OLDER PARTICIPANTS: LESSONS FROM A VACCINE HESITANCY SURVEY

**DOI:** 10.1093/geroni/igae098.2474

**Published:** 2024-12-31

**Authors:** Bryce Van Vleet, Heather Fuller, Andrea Huseth-Zosel

**Affiliations:** North Dakota State University, Fargo, North Dakota, United States; North Dakota State University, Fargo, North Dakota, United States; North Dakota State University, Fargo, North Dakota, United States

## Abstract

Vaccine hesitancy is a critical health risk to older adults who may refuse vaccination for common, preventable, and life-threatening diseases like shingles, pneumonia, COVID-19, and influenza. Despite the importance of understanding vaccine hesitancy among older adults, mistrust in science and hostility towards researchers has been previously documented but it is unclear how these processes impact the research process. Thus, we sought to examine the characteristics and response patterns of volatile survey participants. Data were drawn from a mixed-methods survey about vaccination perceptions and behaviors conducted with 901 community dwelling older adults (65+). Volatile participants (n=59) were identified through their expression of excessive anger (e.g., explicit language, criticism, etc.) or mistrust (e.g., paranoia, accusations, etc.) in open-ended responses. First, linear regression was conducted to identify possible demographic predictors of volatile responses (R2=.063, p<.001). Volatile respondents were significantly more likely to be younger, highly religious, and conservative. Next, chi-square and post-hoc analyses were used to examine differential missingness in volatile participant responses. Volatile participants were more likely to answer questions they likely considered controversial, such as questions about COVID-19 and influenza vaccines. Volatile participants were also less likely to fully complete certain items of vaccine hesitancy and vaccine barrier scales. However, intra-individual response variability analysis did not detect outright deception from volatile participants. Overall, this study highlights methodological challenges in researching vaccine hesitancy among older adults and suggests recommendations to future researchers on conducting studies on politicized topics with skeptical, or even hostile, participants.

